# Development of an automated size-based filtration system for isolation of circulating tumor cells in lung cancer patients

**DOI:** 10.1371/journal.pone.0179744

**Published:** 2017-06-22

**Authors:** Satomi Yagi, Yasuhiro Koh, Hiroaki Akamatsu, Kuninobu Kanai, Atsushi Hayata, Nahomi Tokudome, Keiichiro Akamatsu, Katsuya Endo, Seita Nakamura, Masayuki Higuchi, Hisashige Kanbara, Masanori Nakanishi, Hiroki Ueda, Nobuyuki Yamamoto

**Affiliations:** 1 Medical Business Unit, Hitachi Chemical Co., Ltd., Ibaraki, Japan; 2 Third Department of Internal Medicine, Wakayama Medical University, Wakayama, Japan; Seoul National University College of Pharmacy, REPUBLIC OF KOREA

## Abstract

Circulating tumor cells (CTCs), defined as tumor cells circulating in the peripheral blood of patients with solid tumors, are relatively rare. Diagnosis using CTCs is expected to help in the decision-making for precision cancer medicine. We have developed an automated microcavity array (MCA) system to detect CTCs based on the differences in size and deformability between tumor cells and normal blood cells. Herein, we evaluated the system using blood samples from non-small-cell lung cancer (NSCLC) and small-cell lung cancer (SCLC) patients. To evaluate the recovery of CTCs, preclinical experiments were performed by spiking NSCLC cell lines (NCI-H820, A549, NCI-H23 and NCI-H441) into peripheral whole blood samples from healthy volunteers. The recovery rates were 70% or more in all cell lines. For clinical evaluation, 6 mL of peripheral blood was collected from 50 patients with advanced lung cancer and from 10 healthy donors. Cells recovered on the filter were stained. We defined CTCs as DAPI-positive, cytokeratin-positive, and CD45-negative cells under the fluorescence microscope. The 50 lung cancer patients had a median age of 72 years (range, 48–85 years); 76% had NSCLC and 20% had SCLC, and 14% were at stage III disease whereas 86% were at stage IV. One or more CTCs were detected in 80% of the lung cancer patients (median 2.5). A comparison of the CellSearch system with our MCA system, using the samples from NSCLC patients, confirmed the superiority of our system (median CTC count, 0 versus 11 for CellSearch versus MCA; *p* = 0.0001, *n* = 17). The study results suggest that our MCA system has good clinical potential for diagnosing CTCs in lung cancer.

## Introduction

Circulating tumor cells (CTCs), defined as tumor cells circulating in the peripheral blood of cancer patients, are expected to be a potential prognosis marker for cancers [1–3]. These cells are associated with the prognosis of patients with advanced solid tumors, and reflect characteristics of the respective primary tumor and its metastatic deposits. However, CTCs are rare, occurring at a frequency of approximately 1–10 CTCs per milliliter of cancer patient blood, as opposed to 6 × 10^6^ leukocytes, 4 × 10^9^ erythrocytes, and 2 × 10^8^ platelets. Thus, the technologies for capturing CTCs have received increasing attention in this decade.

Many studies have shown that the appearance of CTCs in peripheral blood is a significant prognostic factor in different types of solid tumors. The number and molecular changes of CTCs may help to predict or monitor treatment responses.

The CellSearch system (Janssen Diagnostics, Raritan, NJ, USA) is currently used as a CTC enumeration device for breast cancer, prostate cancer, and colorectal cancer, having being approved by the US Food and Drug Administration as a medical device [4–6]. The CellSearch system makes use of magnetic beads coated with a monoclonal antibody to capture epithelial cell-adhesion molecules (EpCAMs) on the CTCs. This method is unable to capture CTCs of all cancer types, however, because some CTCs are known to be EpCAM negative [7,8]. Thus, many methods that do not apply anti-EpCAM antibodies have been under development worldwide to address this issue [9–13].

Lung cancer is a leading cause of cancer-related deaths world-wide, accounting for more than 1.59 million deaths per year [14]. However, major progress has been made in molecular targeted therapies for non-small-cell lung cancers (NSCLCs), especially for lung adenocarcinoma [15,16]. Despite the fact that tumors change over time and their genomic and biological characteristics will not stay the same, in most cases, the decision-making in clinical practice over the course of various treatments is based on tumor specimens that were obtained at the time of initial diagnosis. This led us to the urgent need to monitor tumors longitudinally in order to understand their real-time status and to provide the best treatment to our patients. Based on the discussion above, noninvasive methods, especially ones using patient peripheral blood specimens such as CTCs, have been critically needed and intensively investigated. In addition, CTCs have the potential to be used not only for further clarification of the tumor biology but also as a tool for prognosis and/or therapy response prediction. Patients will benefit from this diagnostic technology, because tumor biopsy in lung cancer patients itself is challenging even for the initial diagnosis, mainly because of its anatomic location.

The EpCAM expression-based system seemed to work well for small-cell lung cancers (SCLCs) in previous reports [17,18] but not as well for NSCLCs [6]. Thus, we developed a novel system, the microcavity array (MCA), which does not rely on EpCAM expression and allows the detection of CTCs based on the differences in size and deformability between tumor cells and normal blood cells. We reported the applicability of our developed system in lung cancer patients [19,20]. Recently, we developed a newly automated MCA system that allows much less manual effort and should be more feasible for clinical application. In this present study, we evaluated the performance of the automated CTC detection system using a preclinical spike-in model and blood samples from NSCLC and SCLC patients.

## Materials and methods

### Ethical statement

This study was approved at Wakayama Medical University, in accordance with Institutional Review Board procedures, and all donors provide written informed consent (UMIN000021712).

### MCA system

**Fig 1:**
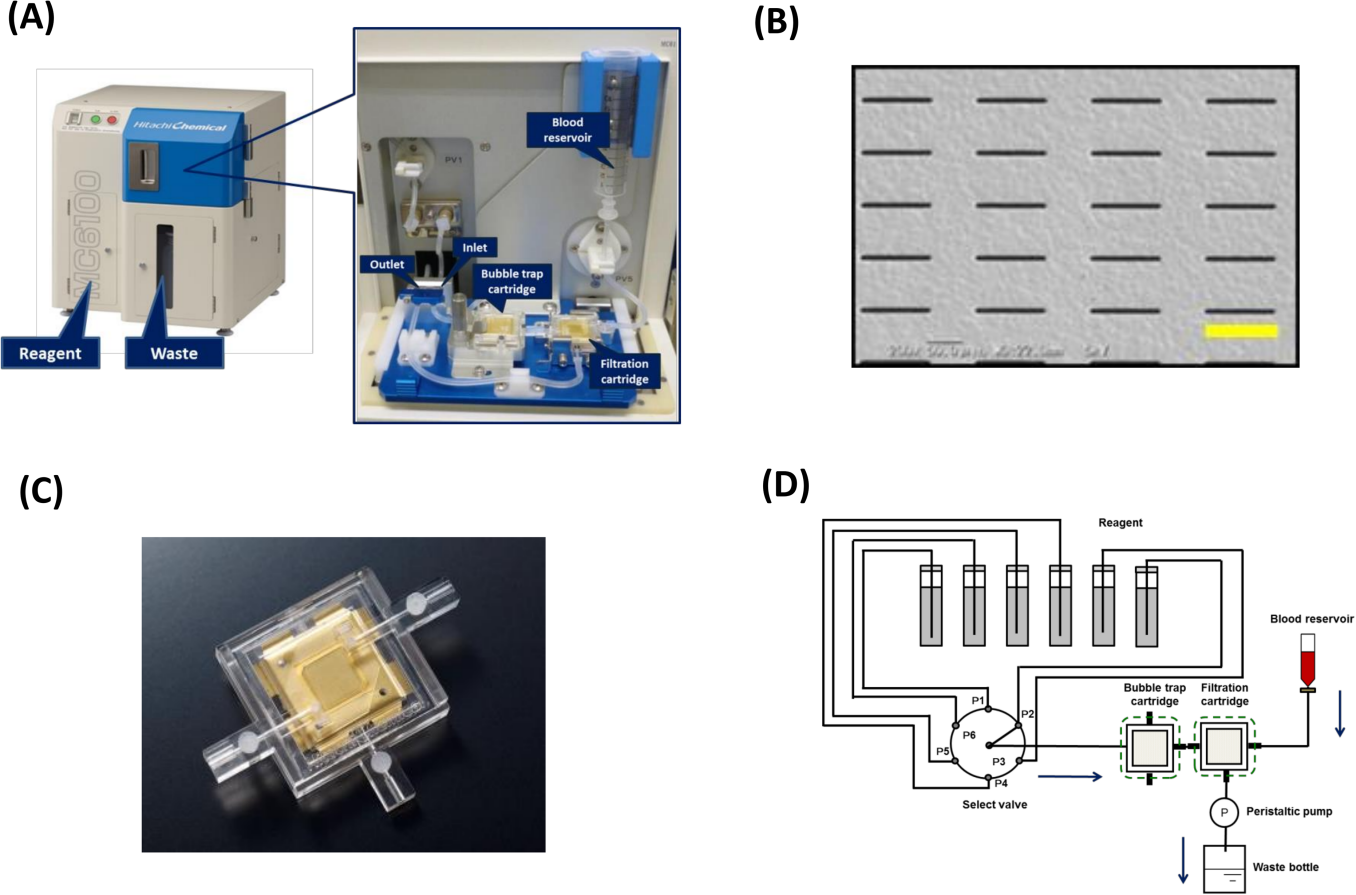
The automated microcavity array (MCA) system. (A) Overview of the MCA system. The system is composed of a blood reservoir, cartridge, and tube. (B) Scanning electron microscope images of a rectangular (8 μm×100 μm) micro metal filter. Scale bar: 100 μm (yellow line). (C) Overview of the cartridge. The cartridge is fabricated from acrylic frames and silicon gaskets. (D) Diagram of the MCA system. The filtration cartridge is connected to two inlets and one outlet. The outlet line is connected to a peristaltic pump to facilitate injection of the blood and each reagent into the cartridge. A bubble trap cartridge is connected to the reagent line to prevent air from entering the filtration cartridge.

The MCA system, which is composed of a blood reservoir, filter-included cartridge, and individual tubes (Fig 1), uses a filtration method with metal filters made of nickel and gold via electroformation. Each fabricated rectangular pore is 8 μm wide and 100 μm long (Fig 1B). The filtration cartridge, made of acrylic frames and silicon gaskets (Fig 1C), is connected to two inlets and one outlet. The outlet line is connected to a peristaltic pump to facilitate the injection of blood and each reagent into the cartridge (Fig 1D).

### Cell culture

Human lung cancer cell lines NCI-H820 (H820), NCI-H441 (H441), A549, and NCI-H23 (H23) were obtained from the American Type Culture Collection (ATCC, Manassas, VA, USA). The H441, A549, and H23 cells were cultured in RPMI-1640 medium (ThermoFisher Scientific, Waltham, MA, USA) containing 10% fetal bovine serum (FBS; Life Technologies, Waltham, MA, USA), at 37°C in a humidified atmosphere containing 5% CO_2_. The H820 cells were cultured in RPMI1640-medium containing 5% FBS, at 37°C in a humidified atmosphere containing 5% CO_2_.

### Cell spiking experiment

The cell lines were cultured for 7–14 days and then cells were harvested with a 0.25% trypsin/ethylenediaminetetraacetic acid (EDTA) (ThermoFisher Scientific) solution at 37°C before the blood spiking experiment. Whole blood (3 mL) was spiked with 100 cells. The blood samples were provided from healthy volunteers at Wakayama Medical University, and written informed consent was obtained from all the donors.

### Clinical study

Human peripheral blood samples were collected from 10 healthy donors and from 50 patients with advanced NSCLC and SCLC at Wakayama Medical University. The blood samples were collected in EDTA-containing blood collection tubes (Becton Dickinson and Company, Franklin Lakes, NJ, USA) to prevent coagulation, and then processed by the MCA system within 3 h. For each donor, 6 mL of blood was collected and 3 mL was used per test, with two replicates tested.

### Tumor cell capturing and staining

Blood samples were added to the reservoir of the MCA system. Subsequently, negative pressure was applied to the whole blood via a peristaltic pump connected to the vacuum line. The sample was filtrated by the metal filter in the cartridge at a flow rate of 600 μL/min. A cell fixation solution and cell staining solution were introduced into the cartridge via the peristaltic pump after washing. The automated staining process is as follows: fixation for 10 min, permeabilization for 10 min, followed by staining for CD45, PD-L1, CK and DAPI for 180 min. 4 min washing is performed between each step and in the final step. Cell fixation and staining reagents were provided from Hitachi chemical company (Ibaraki, Japan).

### Identification and enumeration of CTCs

An image of the entire cell array area was obtained using a fluorescence microscope (Axio Imager M2m; Carl Zeiss, Oberkochen, Germany) integrated with a 10× objective lens and a computer-operated motorized stage, a digital camera (AxioCam 503 mono; Carl Zeiss), and ZEN image acquisition software (Carl Zeiss). We defined a CTC as a DAPI-positive, cytokeratin-positive, and CD45-negative cell. The number of CTCs in 3 mL of peripheral blood was normalized to that in 7.5 mL to compare with the CellSearch system.

### CTC enumeration by the CellSearch system

Twenty-two of the 50 blood samples from patients (17 NSCLC, 5 SCLC) were evaluated for CTCs by using the CellSearch system (Janssen Diagnostics) for direct comparison with our MCA system. Peripheral blood (10 mL) was collected into CellSave Preservative Tubes (Janssen Diagnostics), and measurement with the CellSearch system was performed at GeneticLab Co., Ltd. (Sapporo, Japan).

### Statistical analysis

Statistical analysis was carried out by using GraphPad Prism Version 6 (GraphPad Software, La Jolla, CA, USA).

## Results

### Preclinical study

**Fig 2:**
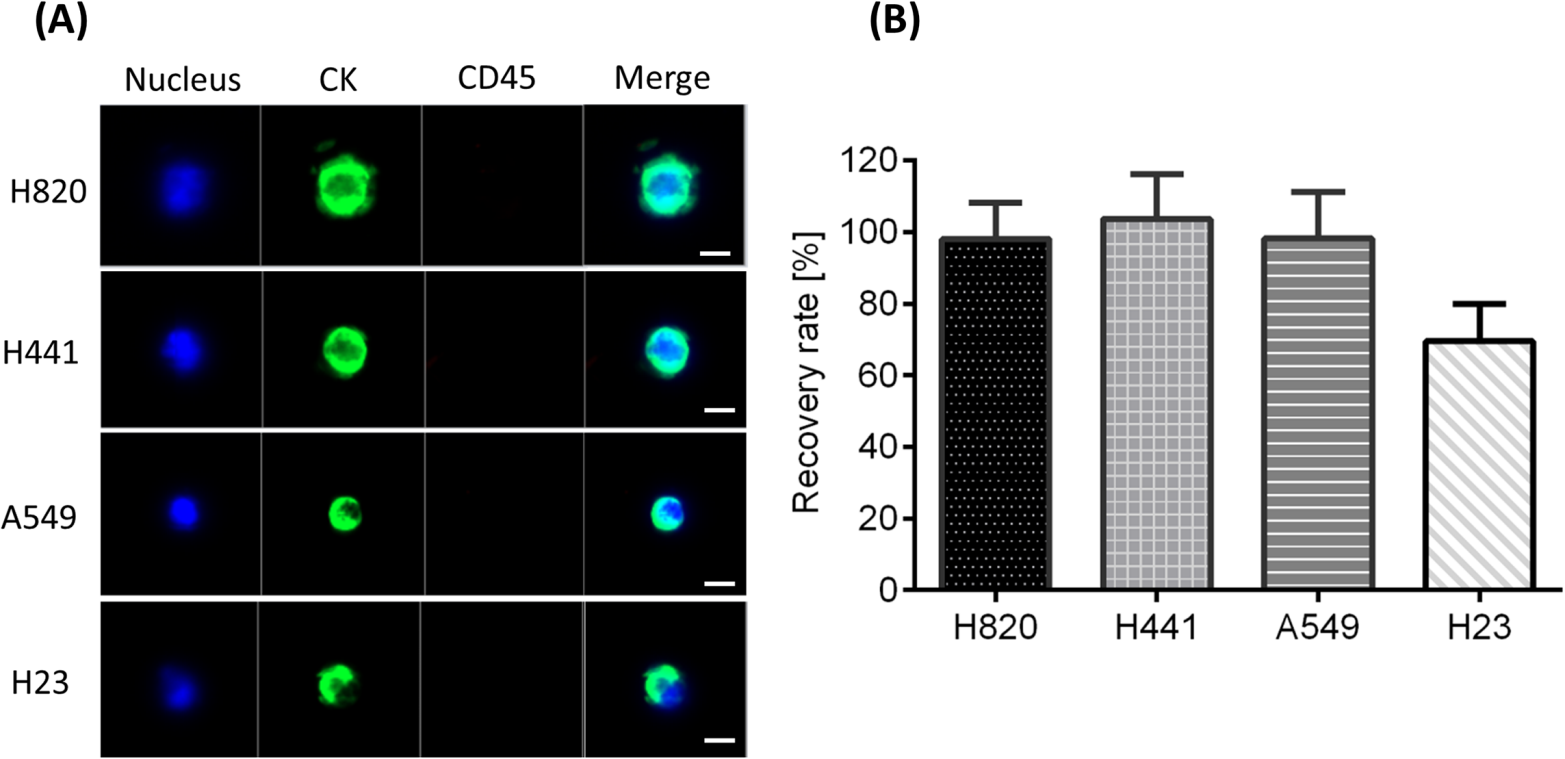
Spike-in experiment for evaluation of the cell recovery rate by the microcavity array (MCA) system. (A) Representative staining images of detected cancer cells. Scale bars: 10 μm. (B) Recovery rates of the lung cancer cell lines by the MCA system.

We evaluated the recovery rate of lung cancer cell lines using the MCA system in spike-in experiments. One hundred cells from the H820, H441, A549, and H23 cell lines were spiked into 3 mL of healthy volunteer blood samples and then processed by MCA assay. Fig 2A shows the fluorescence microscopy images of the cells recovered on the filter. Cells can be defined as CTCs according to the criteria that they are nuclei positive (DAPI stain), cytokeratin positive, and CD45 negative. Fig 2B shows the average recovery rate of each cell line, where that of the H820, H441, and A549 cells exceeded 90%, respectively. The recovery rate of H23 cells was about 70%, which was lower than that of the other cell lines because of the smaller size of H23 cells. Besides this, the expression level of cytokeratin on H23 cells was lower than that on the other cell lines.

### CTC enumeration in lung cancer patients

Between 15 March 2015 and 15 September 2016, 50 lung cancer patients were enrolled into the study and evaluated for CTC counts at Wakayama Medical University (Table 1). Some patient characteristics were as follows: median age 72 years (range, 48–85 years); 64% male, 36% female; 36% never smoker, 64% smoker; 76% NSCLC, 20% SCLC, 4% other; and 14% stage III, 86% stage IV.

**Table 1:** Patient characteristics.

Number of patients:		50
Age: median, (range)	Years	72 (48–85)
Gender: *n*, (%)	Female	18 (36)
	Male	32 (64)
Smoking history: *n*, (%)	Pack-year ≥30	25 (50)
	Pack-year <30	7 (14)
	Never smoker	18 (36)
Histology: *n*, (%)	Adenocarcinoma	30 (60)
	- EGFR* mutated	12 (39)
	- ALK** rearrangement	2 (6)
	Squamous cell	7 (14)
	Small cell	10 (20)
	Others	2 (4)
Performance status: *n*, (%)	0	10 (20)
	1	30 (60)
	≥2	10 (20)
Stage: *n*, (%)	III	7 (14)
	IV	43 (86)
Previous systemic therapies: *n*, (%)	0	35 (70)
	1	8 (16)
	≥2	7 (14)

*EGFR, epidermal growth factor receptor

**ALK, anaplastic lymphoma kinase.

**Fig 3:**
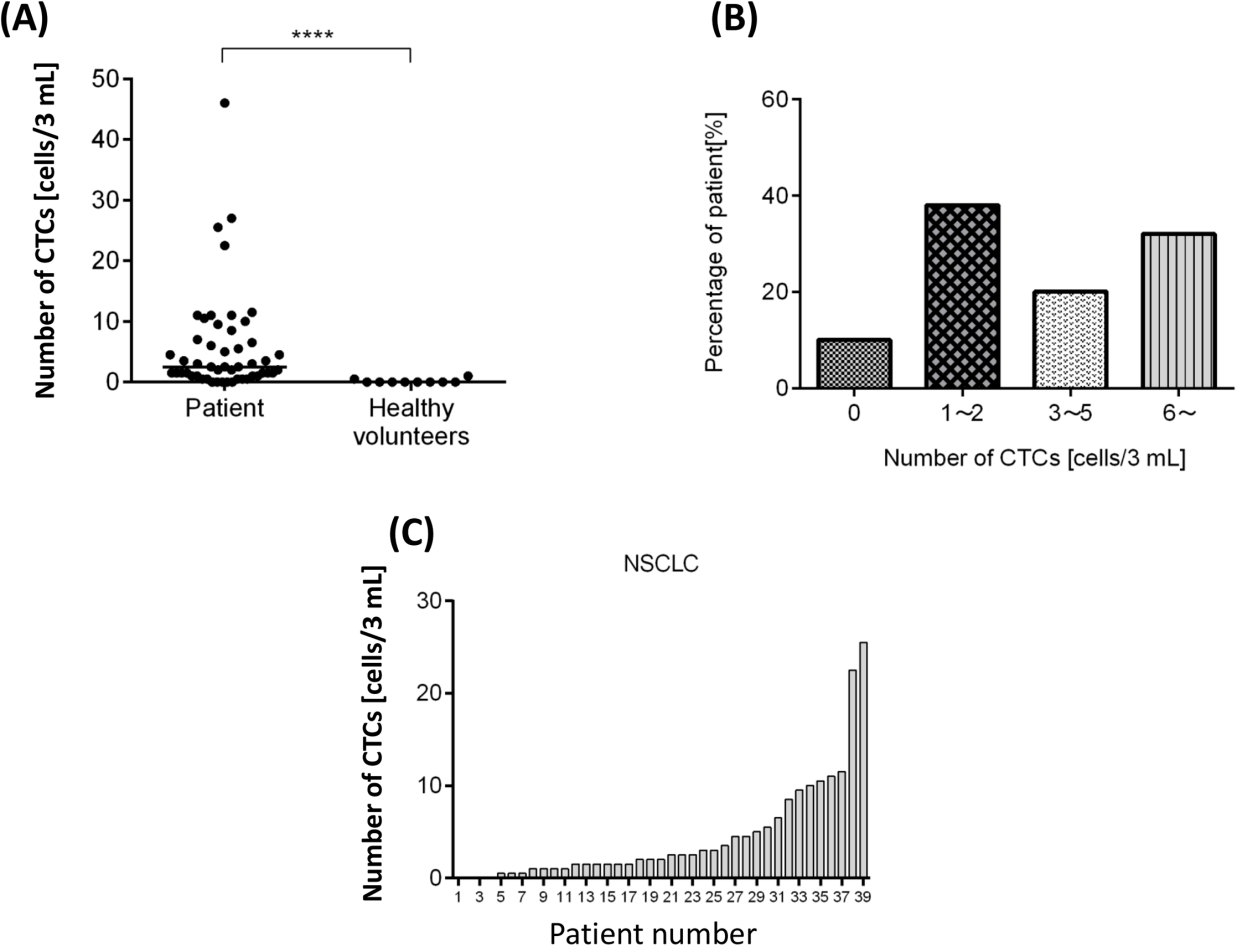
Results of circulating tumor cell (CTC) enumeration in clinical study. (A) Comparison between lung cancer patients and healthy volunteers (*n* = 50 and 10, respectively) in the number of CTCs (median = 2.5 and 0, respectively); ****p < 0.0001. (B) Distribution of CTC count in all patients. (C) CTC count in 37 NSCLC patients.

CTCs were detected in 40 out of the 50 patients (median 2.5; range, 0–46) and more than 5 CTCs were detected in 32% of the patients (Fig 3A and 3B). Of the 10 healthy volunteers, CTCs were detected in only 1 individual, for which the count was 1 cell in 3 mL of blood, suggesting a very low chance of a false-positive event. More CTCs were detected in the lung cancer patients than in the healthy volunteers, with statistical significance (*p* = 0.0001). Fig 3C shows the number of CTCs detected in NSCLC patients.

### Comparison with the CellSearch system

**Fig 4:**
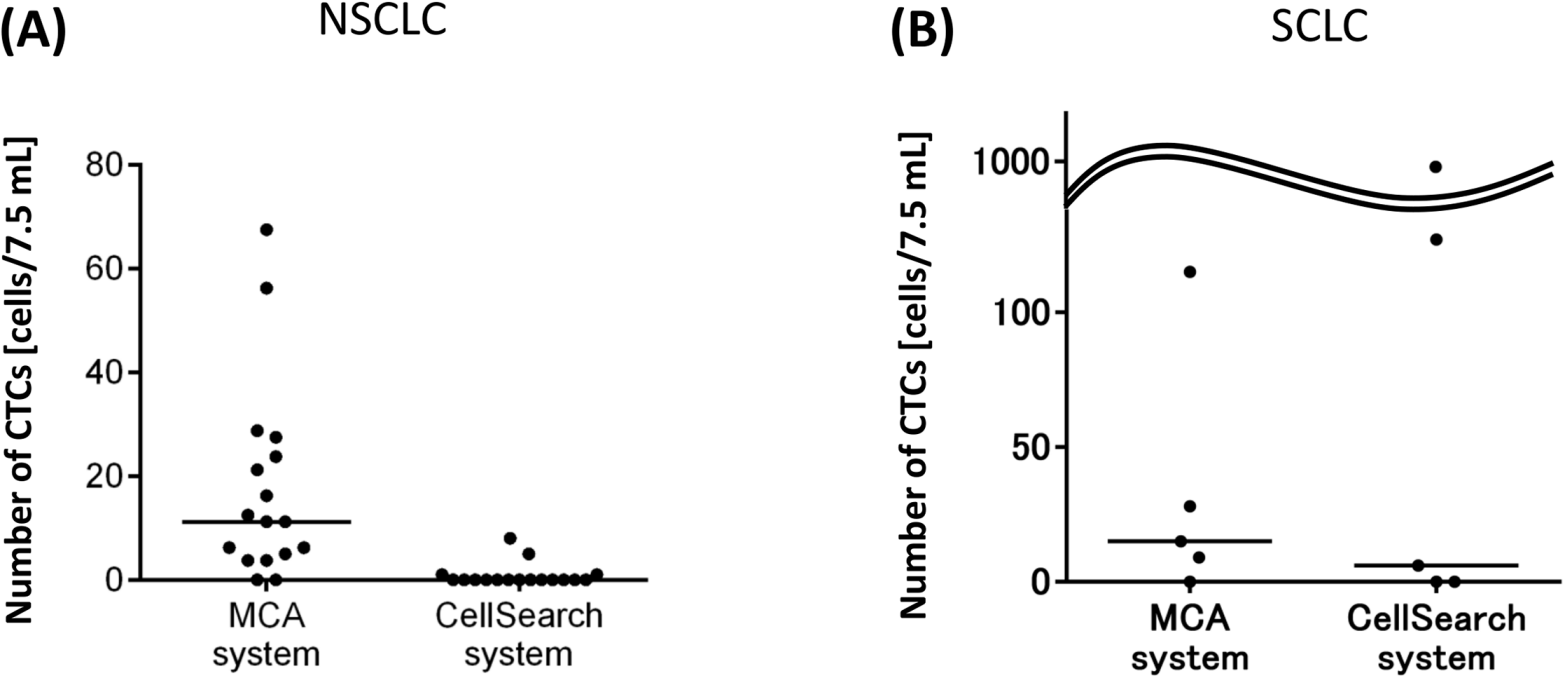
Comparison between the microcavity array (MCA) system and CellSearch system in circulating tumor cell (CTC) count. (A) Counts in NSCLC patients (*n* = 17) (median, 11 versus 0); ***p < 0.0001. (B) Counts in SCLC patients (*n* = 5) (median, 15 versus 6).

We compared the CTC counts obtained by the MCA system and the currently standard CTC measurement system, CellSearch. Fig 4 and Tables 2 and 3 show the number of CTCs in 7.5 mL of whole blood from lung cancer patients. The median CTC count in NSCLC patients was 0 cell for the CellSearch system and 11.25 cells for the MCA system (*n* = 17, each system), revealing the number detected by the MCA system to be significantly higher (*p* < 0.001). On the other hand, in samples from SCLC patients, there was no significant difference in terms of detection sensitivity between the two systems. The current MCA system filter is optimized for capturing CTCs of NSCLC based on the differences in size and deformability between tumor cells and normal blood cells. In other words, a fair number of SCLC CTCs was still detectable, despite the fact that the MCA system was not necessarily optimized for capturing tumor cells in SCLC patients.

**Table 2:** Comparison of circulating tumor cell counts in NSCLC samples detected by the microcavity array (MCA) and CellSearch systems.

	MCA	CellSearch
N1	11	0
N2	28	1
N3	6	0
N4	16	0
N5	0	0
N6	4	5
N7	29	0
N8	0	0
N9	21	8
N10	4	0
N11	11	0
N12	5	1
N13	6	0
N14	13	0
N15	56	0
N16	24	0
N17	68	0

N, Non-small-cell lung cancer.

**Table 3:** Comparison of circulating tumor cell counts in SCLC samples detected by the microcavity array (MCA) and CellSearch systems.

	MCA	CellSearch
S1	28	127
S2	0	0
S3	9	6
S4	115	981
S5	15	0

S, Small-cell lung cancer.

### Clinicopathologic correlation

**Fig 5:**
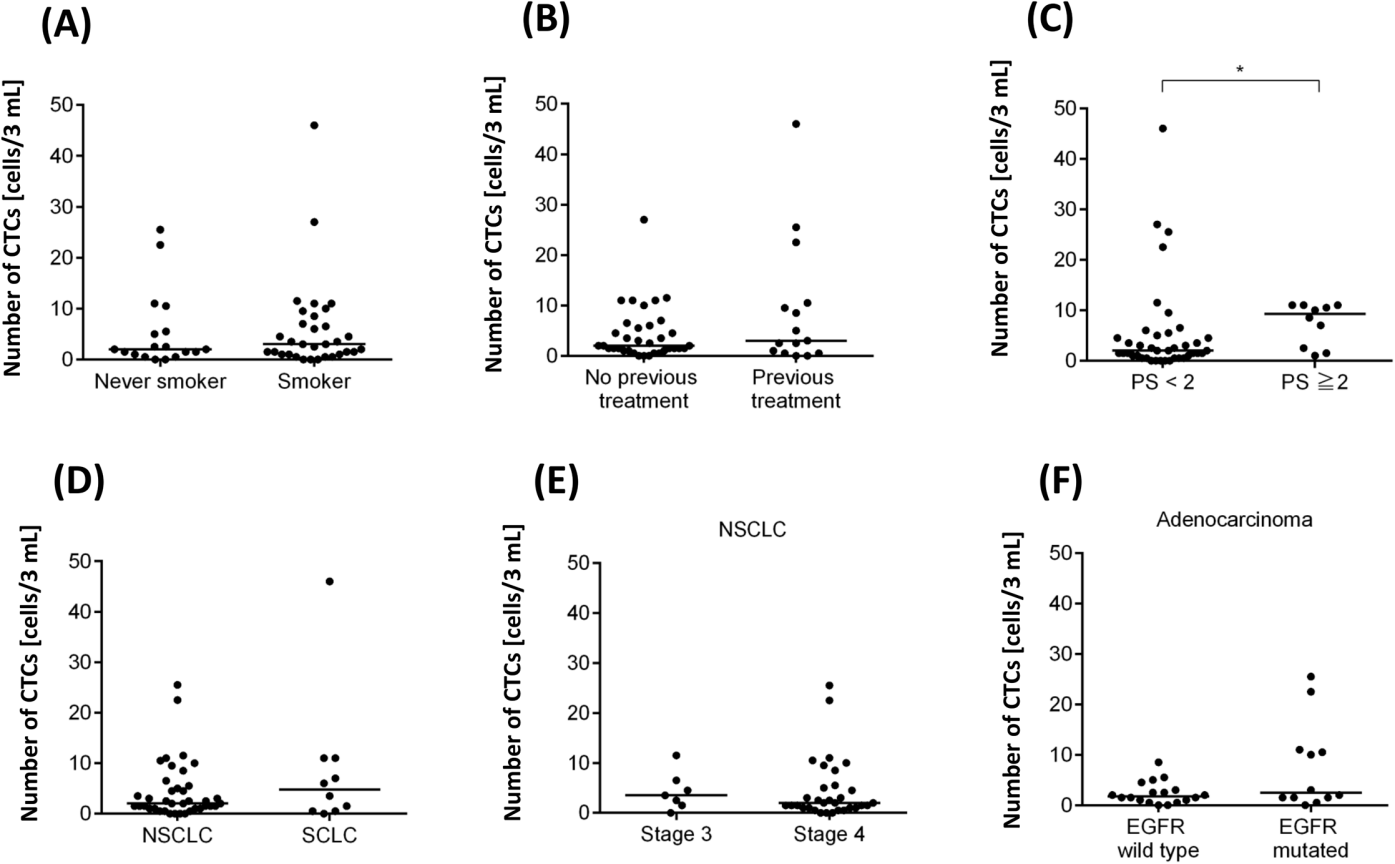
Clinicopathologic correlation of circulating tumor cell (CTC) counts by MCA in lung cancer patients. (A) Never smokers (n = 18) versus smokers (n = 32). (B) Patients with no previous treatment (n = 35) versus patients with previous treatment (n = 15). (C) Performance status (PS) <2 (n = 40) versus PS ≥2 (n = 10). (D) NSCLC (n = 39) versus SCLC (n = 10). (E) Stage III (n = 7) versus stage IV (n = 32) in NSCLC patients. (F) EGFR wild-type adenocarcinoma (n = 18) versus EGFR-mutated adenocarcinoma (n = 12).

We correlated the number of CTCs detected by MCA with 6 clinicopathologic factors; namely, smoking history, previous treatment history, performance status (PS), histology, tumor stage, and epidermal growth factor receptor (EGFR) mutational status (Fig 5). Most of the correlation analyses did not turn out to be positive, except for PS (Fig 5C). As shown in Fig 5C, the median CTC count in patients with PS <2 was 2 cells, whereas that in patients with PS ≥2 was 9.3 cells. This significantly higher CTC count in the patients with PS ≥2 (*p < 0.05) suggests that the tumor may be more widespread in the blood stream in those with a poorer PS. Fig 5E shows the comparison of the CTC numbers between stage III and stage IV NSCLCs, where the former group had a median count of 3.5 cells and the latter group had a median count of 2 cells.

## Discussion

In this study, we evaluated the detection of CTCs from whole blood of lung cancer patients by the newly automated MCA system and found that we could detect as many CTCs as we did in previous reports [19,20]. We also confirmed that the MCA system was superior to the CellSearch system (which is still only under FDA-approved testing status to date) in NSCLC patients. These results suggest that CTCs can be potentially used for lung cancer management in the era of precision medicine, thanks to the improved sensitivity of the MCA system.

Although CTC diagnosis has been intensively investigated and explored to realize real-time monitoring and earlier decision-making regarding solid tumors, such as breast and prostate cancers, this was not necessarily the case for lung cancer. For those studies, the CellSearch system was applied as the diagnostic tool, which works for breast and prostate cancers. However, it has been reported that CellSearch does not work as well for NSCLCs [6]. One of the reasons would be that the CellSearch system depends on EpCAM expression for capturing the tumor cells, which is not necessarily expressed by all CTCs. In order to improve CTC detection sensitivity, it is essential to incorporate enrichment methods other than EpCAM-based ones. As our MCA system is not dependent on EpCAM expression for tumor cell enrichment, it potentially allows us to detect CTCs of cancer types with low and negative EpCAM expression.

Though the CTC detection sensitivity was improved with MCA system, we did not observe much clinicopathologic correlation in this study. One of the reasons for this is that the study was conducted in a relatively small cohort to evaluate the feasibility of the automated MCA system. For example, SCLC patients are supposed to harbor more CTCs than NSCLC [21], but we had only 10 SCLC patients enrolled in the study, resulting in insufficient statistical power. However, patients with poorer PS (≥2) still harbored significantly more CTCs than those with PS 0 or 1 (Fig 5C). This observation strongly supports the previous reports that CTC count can be a prognostic factor in lung cancer patients [6,18] because patients with poor PS usually have poor prognosis. To further clarify the correlation between CTC count and clinicopathologic factors, it is necessary to conduct a study in a larger cohort in the future.

Recently, results of CTC detection with filtering methods such as CellSieve have been reported [22,23]. CellSieve consists of a polycarbonate filter and syringe folder, and CTCs can be captured automatically. However, cell fixation and staining for CTC detection need to be performed manually. Souza et al. reported the results of CTC detection in colorectal cancer patients by ScreenCell, another filtering system [9–11,24]. Most of the filtering devices in common are provided solely for CTC capture, and the method and criteria for CTC recognition need to be established by researchers. On the other hand, our MCA system covers all the steps from capturing the cells to staining them, resulting in much less manual effort and less turnaround time. Our system also shows data reproducibility. All these factors are important for clinical application in the future.

CTCs can be used for various clinical applications as previously reported [4–6] and they have advantages over circulating tumor-derived DNA (ctDNA), which is currently approved for EGFR mutation testing [25]. ctDNA is very useful for genomic analyses, such as mutation detection, something that CTCs may not be necessarily easy to work with mainly because of the limited number of retrieved cells and the multiple processes from DNA extraction to sequencing. However, CTCs definitely have unique advantages over ctDNA. Most importantly, CTCs can be used for evaluating protein expression on them, which can be a target of cancer therapeutics. Recently, immune checkpoint blockade with programmed death 1 (PD-1)/PD-ligand 1 (PD-L1) inhibitors was demonstrated to be efficacious for lung cancer patients [26], and PD-L1 expression on tumor tissues could be a predictive biomarker of clinical benefit in NSCLC patients. However, it is still challenging to obtain a tumor biopsy from lung cancer patients, especially a real-time biopsy at the point when the treatment is initiated. We believe that CTCs can be used as an alternative material instead of tumor tissues for PD-L1 expression evaluation. Our group and others are presently detecting PD-L1 expression on CTCs [27,28].

There are some challenges to be addressed with our MCA system. Our results indicate that we cannot detect as many CTCs as we expected in SCLC patients. The capture principle of our MCA system is based on the difference in cell size between tumor cells and normal blood cells, and it is inevitable that we lose tumor cells of smaller size such as SCLC cells. Although we had previously tackled this and modified the filter, achieving better detection sensitivity than that of CellSearch, more improvement is clearly needed. For future large-scale multicenter clinical studies, we have to develop a preservative device that allows a larger window for sample processing. In this study, most of the blood samples were processed within 3 h after the blood had been drawn, and it can be done up to 6 h. We are in the process of establishing a method that allows a longer window of up to 72 h, using preservative blood collection tubes, and this will be applied for future clinical studies, including the evaluation of PD-L1 on CTCs.

In conclusion, we have evaluated the performance of our newly developed automated MCA system in detecting CTCs in whole blood from lung cancer patients. The results were promising and suggest that the MCA system has clinical potential for CTC diagnosis in lung cancer. Nevertheless, more investigation on a larger scale is warranted.
